# Associations between interpersonal violence and cigarette smoking, e-cigarette use, and dual use among Mexican adolescent students

**DOI:** 10.3389/fpubh.2025.1516135

**Published:** 2025-02-12

**Authors:** Rosibel Rodríguez-Bolaños, Evangelina Díaz-Andrade, Paula Ramírez-Palacios, Anabel Rojas-Carmona, Katia Gallegos-Carrillo, Inti Barrientos-Gutiérrez, Lizeth Cruz-Jiménez, Dèsirée Vidaña-Pérez, Edna Arillo-Santillán, James F. Thrasher

**Affiliations:** ^1^Department of Reproductive Health, Population Health Research Center, National Institute of Public Health, Cuernavaca, Morelos, Mexico; ^2^Department of Research, Health Services of Jalisco, Secretary of Health, Health Region VI, Ciudad Guzmán, Jalisco, Mexico; ^3^Southern Region University Center, University of Guadalajara, Ciudad Guzmán, Jalisco, Mexico; ^4^Evaluation and Survey Research Center, National Institute of Public Health, Cuernavaca, Morelos, Mexico; ^5^Faculty of Nursing, University of the Sea, Puerto Escondido, Oaxaca, Mexico; ^6^Faculty of Medicine, Graduate Program in Public Health, University Federal of Ceará, Fortaleza, Ceará, Brazil; ^7^Epidemiology and Health Services Research Unit, Mexican Social Security Institute, Cuernavaca, Morelos, Mexico; ^8^Department of Health Promotion, Education & Behavior, University of South Carolina, Columbia, SC, United States; ^9^Department of Tobacco Control, Population Health Research Center, National Institute of Public Health, Cuernavaca, Morelos, Mexico

**Keywords:** violence, neglect, tobacco use, electronic cigarette, adolescents

## Abstract

**Objective:**

To assess associations between experienced violence and the use of combustible cigarettes, e-cigarettes, and of both products (“dual use”) among adolescent students from Mexico.

**Methods:**

Data comes from an online survey among 3,046 adolescents (12–19 years) conducted between September and December 2021. Students reported experiences of neglect, physical, psychological, and sexual violence perpetrated by household members, and digital violence (by anyone) in the previous 12 months, as well as current (last 30 day) use of cigarettes and e-cigarette. In multinomial logistic models, exclusive cigarette use, exclusive e-cigarette use, and dual use (reference = no use) were regressed on experienced violence and covariates.

**Results:**

The prevalence of exclusive use of cigarettes was 1.4%, exclusive e-cigarette use was 6.1, and 2.4% for dual use. Almost half (46.9%) of adolescents reported having been the target of physical violence, followed by psychological violence (42.6%), neglect (34.9%), digital violence (12.3%), and sexual violence (5.2%). In adjusted multinomial models, adolescents who experienced physical violence (Adjusted Relative Risk Ratios: ARRR = 2.28, 95% CI [1.05–4.96]) were more likely to exclusively smoke cigarettes. Adolescents were also more likely to exclusively use e-cigarettes if they had been targeted by psychological or digital violence (ARRR = 1.55, 95% CI [1.05–2.29] and ARRR = 1.69, 95% CI [1.12–2.54], respectively). Experience of physical, digital, neglect, and sexual violence were positively associated with dual use.

**Conclusion:**

Experiences of violence may increase the likelihood of tobacco use, particularly dual use. Prevention programs may need to include the family environment to reduce violence.

## 1 Introduction

Violence is a significant global public health concern due to its detrimental effects on both physical and mental development. During the pandemic, several countries including China, England, the United States of America, France and Brazil reported an increase in domestic and gender-based violence, especially toward women, children and adolescents (1). In five Latin American countries, including Mexico, an increase in domestic physical altercations was reported (2). Adolescents who experience violence encounter a multitude of challenges in their personal development, as well as in their interpersonal and social relationships.

Exposure to violence within the family has been demonstrated to influence in nicotine product use among adolescents (3, 4). Physical, psychological and sexual violence in the family have been associated with an elevated risk of consuming cigarettes, alcohol (5), and other psychoactive substances (6). Additional studies have found youth who experience various forms of neglect and digital violence are also more likely to use cigarettes and e-cigarettes than youth who do not have these experiences (7, 8). To date, however, no studies have identified the specific types of violence associated with e-cigarette use or “dual use” of both cigarettes and e-cigarettes.

In many countries, smoking among adolescents has declined while e-cigarette use has increased (9). The rise of youth e-cigarette poses significant public health concerns because they can adversely impact the developing brain during adolescence, a period when risk-taking behaviors are prevalent (10). Research has identified a number of risk factors for adolescent e-cigarette use, such as being male, older, belonging to a sexual or gender minority, traumatic childhood experiences, peer influence, and alcohol and other substance use (11–13). A comprehensive understanding of the risk factors for e-cigarette use can help identify targets for prevention strategies.

### 1.1 Study context

Jalisco is a Mexican state where adolescents aged 12–29 comprise about a third (31.2%) of the population (14), making it one of the states with the highest proportion of adolescents in the country. Before the COVID-19 pandemic, 60.6% of women aged 15–29 reported experiencing some form of violence in the last 12 months. In 2022, Jalisco ranked among the top 10 Mexican states (out of 32) with the highest rates of femicides and reports of family violence (15). Moreover, in a study conducted with adolescents in Ciudad Guzmán, in southern Jalisco, 5.0% reported interpersonal violence experienced in their lifetime (16, 17), underscoring the severity of this issue.

In Mexico, the 2021 National Health and Nutrition Survey (ENSANUT-2021) (18) estimated that 2.7% of adolescents smoked and 3.5% used e-cigarettes. Prior to the pandemic, the prevalence of cigarette smoking (3.7%) and e-cigarette use (1.7%) among adolescents in Jalisco was similar to these national estimates (19). Prior research on urban Mexican adolescents found that males, those with greater family wealth, current smokers, those with higher “technophilia” (i.e., pleasure from using new technologies), and those whose family members used cigarettes and e-cigarettes were more likely to use e-cigarettes (20, 21). Furthermore, studies in other countries have found that parental rules regarding media use in the home, has been demonstrated to play a significant role in the initiation of cigarette and e-cigarette use (22, 23). To our knowledge, there is only one study that has examined the association between neglect and sexual violence and cigarette smoking (24). However, not for all five types of violence and the use of any nicotine product among Mexican adolescents.

The objective of this study was to evaluate the association between exposure to violence and the exclusive use of combustible cigarettes, e-cigarettes, and dual use (consumption of both cigarettes and e-cigarettes) among Mexican middle and high school students in Jalisco, Mexico, during the COVID-19 pandemic.

## 2 Materials and methods

### 2.1 Study design and population

The annual Mental Health, Addictions, and Violence Survey collects cross-sectional data to assess critical issues in these areas. The questions used in this study were primarily adapted from the Global Youth Tobacco Survey (GYTS), a validated instrument widely applied in research involving adolescents in Mexico (25). A convenience sample of public middle schools (*n* = 51) and high schools (*n* = 19) from 16 municipalities in southern Jalisco participated. The survey was anonymous was launched online via Google Forms from September to December 2021.

Informed consent and assent were obtained prior to data collection via Google Forms. School director provided consent to conduct the study within their educational institutions during the pandemic. This facilitated the implementation of the study in a controlled and accessible environment. Recruitment was carried out using two methods: (a) school director shared a link to the online questionnaire directly with students, and (b) a team of health promoters from the Ministry of Health visited schools in person to invite students to participate. The questionnaire was accessible via cell phones, tablets, or computers, and in some cases, students were directed to computer rooms. Students who agreed to participate completed a 103-item multiple-choice questionnaire. Additionally, all participants were given information about the 075 helpline and mental health and addiction care centers.

A total of 3,215 students entered the survey portal, 126 declined to participate, and 43 failed to meet the age criteria. A total of *n* = 3,046 adolescents from 12 to 19 years old completed the survey.

### 2.2 Measurements

#### 2.2.1 Dependent variable

Current cigarette use was determined by asking students: “During the past 30 days, how many days did you smoke cigarettes?,” with those who reported smoking at least once defined as current smokers. Similarly, for current e-cigarette use, students were asked: “During the past 30 days, how many days did you use e-cigarettes?” Those who reported using e-cigarettes at least once in the prior 30 days were defined as current users. These two categories were used to divide the sample into (1) non-current users (of neither product); (2) exclusive cigarette users (only smoked); (3) exclusive e-cigarette users (only used e-cigarettes); and (4) dual users, used both cigarette and e-cigarettes over the prior month.

#### 2.2.2 Violence

We collected information on five types of violence experienced in the past 12 months (26), using validated measures that capture key areas of violence that children and adolescents experience. Students reported on four types of violence that household members had perpetrated on them: (1) physical violence: (a) “Have you had any type of objects such as shoes, kitchen utensils, or furniture thrown at you, whether or not it hit you?” (b) “Have you been slapped anywhere on your body?” (c) “Have you been burns with an iron, the stove, a match or cigarette or any liquid or another hot object on your body?”; (2) psychological violence: (a) “Has anyone referred to you with rude or aggressive words that have made you feel bad?” (b) “Have you had been made fun of due to your physical characteristics, or your knowledge, or your way of thinking, acting, and feeling?” (c) “Have you been humiliated?”; (3) sexual violence: (a) “Have you been sexually harassed or forced to let yourself be touched or caressed against your will?” (b) “Have you been forced to have sexual intercourse against your will, without or with the use of physical force?”; (4) Neglect: (a) “Have you been tied you up to prevent you from going out or doing what you want to do?”; (b) Have you been prevented from going to the doctor or had your state or health condition neglected when you needed care?”; (c) “Have your diet, clothing, recreation, or education been restricted at home?.” Answering “yes” to any of the questions within any domain was regarded as having experienced that type of violence. A question regarding students’ experiences of digital violence (i.e., “Have you received any type of violence or harassment through the internet/digital social media?”) referred to perpetrators of any type, not just family members.

#### 2.2.3 Covariates

Questions on sociodemographic included sex, sexual orientation (27) [responses: heterosexual, gay/lesbian/bisexual/other (LGB), do not know], age (two categories: 12–14 years and 15–19 years), family structure [adolescent is living with: both parents, one parent, other people (e.g., grandparents, uncles/aunts, cousins, siblings, partners)], and being employed (Yes/No). Wealth was measured with the Family Affluence Scale (FAS) (28), an index comprising four items: (a) how many cars or vans does your family own? (0/1/2 or more), (b) do you have a room to yourself? (0/1), (c) during the past 12 months, how many times did you go on vacation with your family? (0/1/2/3 or more), and (d) how many computers does your family have? (0/1/2/3 or more), with scores summed (range = 0–9) so that higher scores indicated greater family wealth. Scores were re-coded into three categories: low (0–2 points), medium (3–5 points), and high (6–9 points).

Students reported on cigarette and e-cigarette use among family members who live with them and among their five closest friends, with responses dichotomized to indicate the use of each product (Yes/No) separately for family and friends (29). Parental rules about media use were evaluated through four questions: (1) videogame use, (2) time spent on the Internet, (3) what you see on the Internet, and (4) what you watch on TV, resulting a variable with three categories: None = 0 of 4; some rules = 1–2 of 4 rules; many rules = 3–4 of 4 rules (30).

### 2.3 Statistical analysis

A descriptive analyses characterized the distribution of students in each category of categorical variables. Proportions of each study variable were calculated for each category of use (past 30-day) of combustible cigarettes and e-cigarettes: (1) non-current user (reference category), (2) exclusive cigarette user, (3) exclusive e-cigarette users, and (4) dual user. We also estimated multinomial logistic models, regressing tobacco product use (reference = students who did not use either product) on victimization by each of the five types of violence (physical, psychological, neglect, sexual, and digital), as well as all covariates. Assessment of potential collinearity among independent variables indicated no concerns (i.e., all variance inflation factors < 2.0). Analyses were performed using STATA 15 (31).

## 3 Results

**Figure 1:**
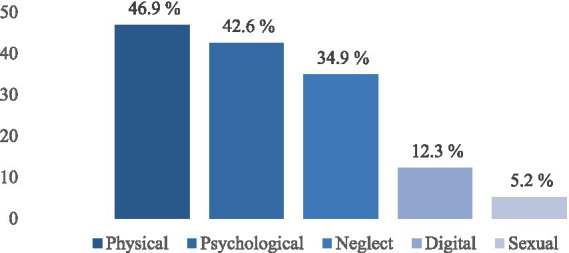
Experienced violence interpersonal among middle and high school students, Jalisco 2021.

Of the 3,046 participants (Table 1), more than half were female (57.4%), most identified as heterosexual (64.5%), and the majority were aged between12 and 14 years (78.0%). About half (51.3%) of participants resided with both parents, 29.5% were employed, and 34.6% had low FAS. Almost half of the adolescents surveyed reported having been the target of physical violence (46.9%), with 42.6% reporting psychological violence, 34.9% neglect, 12.3% digital violence and 5.2% sexual violence (Figure 1).

**Table 1:** Characteristics of the among middle and high school students, Jalisco 2021.

Variables	Total (*n* = 3,046)	
	*n*	%
Sex		
Male	1,299	42.7
Female	1,747	57.4
Age years		
12–14	2,377	78.0
15–19	669	22.0
Sexual orientation		
Heterosexual	1,966	64.5
Gay/lesbian/bisexual/others	257	8.4
Do not know	823	27.0
Family structure. Living with:		
Both parents	1,562	51.3
One of the parents	949	31.2
Other people*	535	17.6
Family affluence scale		
Low	1,054	34.6
Medium	1,366	44.9
High	626	20.6
Employed		
Yes	897	29.5
Parental rules about videogames, Internet and TV use**		
None	512	16.8
Some	865	28.4
Many	1,669	54.8
Current use of nicotine products		
None	2,744	90.1
Exclusive cigarette user	42	1.4
Exclusive e-cigarette user	186	6.1
Dual user	74	2.4
Parental smoking cigarette		
No	1,424	46.8
Yes	1,622	53.3
Friends smoking cigarette		
None	2,344	76.9
One or more	702	23.1
Parental e-cigarette use		
No	2,750	90.3
Yes	296	9.7
Friends e-cigarette use		
None	2,304	75.6
One or more	742	24.4

*Include both grandparents (0.92%), one grandparent (3.48%), aunts/uncles or both (8.93%), cousin (1.12%), older brothers/sister (2.36%) and partners (0.79%).

**Parental rules about media use: None = 0 of 4 rules, some = 1–2 of 4 rules, many = 3–4 of 4 rules.

### 3.1 Prevalence and factors associated with current nicotine product users

The prevalence of exclusive current use of combustible cigarettes was 1.4%, exclusive e-cigarette use was 6.1, and 2.4% used both products (i.e., “dual use”) (Table 1).

In adjusted multinomial models (see Table 2), exclusive cigarette use was higher among adolescents who resided with one (vs. both) of their parents (Adjusted Relative Risk Ratios: ARRR = 2.46, 95% CI [1.17–5.18]), had one or more friends who smoked (ARRR = 6.78, 95% CI [3.18–14.43]), and had experienced physical violence (ARRR = 2.28, 95% CI [1.05–4.96]). Conversely, adolescents who reported some and many regulations of parental rules around media use (vs. none) were less likely to exclusively smoke (ARRR = 0.37, 95% CI [0.17–0.80]).

**Table 2:** Prevalence and factors associated with current nicotine products users among middle and high school students, Jalisco 2021.

		Current user nicotine products					
Variables	None	Exclusive cigarettes user		Exclusive e-cigarettes user		Dual user	
	*n* = 2,744	*n* = 42		*n* = 186		*n* = 74	
	%	%	ARRR (95% CI)	%	ARRR (95% CI)	%	ARRR (95% CI)
Type of violence							
Physical							
No	93.8	0.7	Ref	4.6	Ref	1.0	Ref
Yes	85.9	2.2	2.28 (1.05–4.96)*	7.8	0.97 (0.67–1.39)	4.1	1.99 (1.05–3.78)*
Psychological							
No	94.4	0.9	Ref	3.5	Ref	1.3	Ref
Yes	84.3	2.1	1.09 (0.51–2.30)	9.6	1.55 (1.05–2.29)*	4.0	0.93 (0.49–1.73)
Neglect							
No	93.1	1.0	Ref	4.6	Ref	1.4	Ref
Yes	84.5	2.2	1.47 (0.76–2.82)	8.9	1.35 (0.96–1.89)	4.4	1.97 (1.15–3.38)*
Digital							
No	92.1	1.2	Ref	5.0	Ref	1.7	Ref
Yes	75.5	2.7	1.17 (0.52–2.66)	14.1	1.68 (1.12–2.54)*	7.7	1.93 (1.06–3.52)*
Sexual							
No	91.1	1.2	Ref	5.8	Ref	1.9	Ref
Yes	71.1	4.4	1.74 (0.68–4.50)	12.0	0.97 (0.53–1.78)	12.6	2.65 (1.30–5.40)**
Sex							
Male	90.9	1.3	Ref	5.3	Ref	2.5	Ref
Female	89.5	1.7	0.99 (0.50–1.97)	6.7	0.93 (0.65–1.33)	2.4	0.56 (0.32–0.99)*
Age years							
12–14	92.2	1.3	Ref	4.9	Ref	1.6	Ref
15–19	82.7	1.6	0.65 (0.30–1.42)	10.5	1.31 (0.90–1.91)	5.2	1.78 (1.01–3.14)*
Sexual orientation							
Heterosexual	91.5	1.0	Ref	5.5	Ref	2.1	Ref
Gay/lesbian/bisexual/others	77.4	2.3	1.91 (0.69–5.27)	12.8	1.69 (1.04–2.74)*	7.4	2.34 (1.18–4.63)*
Do not know	90.8	2.1	2.39 (1.19–4.82)	5.5	1.28 (0.86–1.90)	1.7	1.23 (0.62–2.42)
Family structure, living with							
Both parents	92.4	0.8	Ref	4.7	Ref	2.1	Ref
One of the parents	87.8	2.1	2.46 (1.17–5.18)*	7.4	1.52 (1.05–2.21)*	2.7	1.25 (0.70–2.24)
Other people	87.5	1.9	2.21 (0.92–5.32)	7.9	1.47 (0.95–2.27)	2.8	1.13 (0.56–2.26)
Family Affluence Scale							
Low	93.1	1.2	Ref	4.1	Ref	1.6	Ref
Medium	89.9	1.5	1.00 (0.48–2.08)	6.1	1.16 (0.77–1.75)	2.6	1.10 (0.58–2.10)
High	85.5	1.4	1.18 (0.48–2.88)	9.6	1.71 (1.09–2.69)*	3.5	1.49 (0.73–3.06)
Employed							
No	92.9	1.0	Ref	4.5	Ref	1.5	Ref
Yes	83.3	2.2	1.64 (0.85–3.15)	9.9	1.68 (1.20–2.37)**	4.6	1.69 (0.98–2.89)
Parental rules about media use							
None	83.0	2.9	Ref	9.8	Ref	4.3	Ref
Some	88.7	1.3	0.41 (0.18–0.95)*	7.2	0.69 (0.45–1.07)	2.9	0.69 (0.36–1.35)
Many	93.0	1.0	0.37 (0.17–0.80)*	4.4	0.65 (0.42–1.01)	1.6	0.67 (0.34–1.32)
Parental smoking cigarette							
No	94.3	0.9	Ref	3.8	Ref	1.0	Ref
Yes	86.4	1.8	1.23 (0.61–2.51)	8.1	1.26 (0.87–1.81)	3.7	2.03 (1.05–3.91)*
Friends smoking cigarette							
None	95.7	0.6	Ref	3.2	Ref	0.5	Ref
One or more	71.4	3.9	6.78 (3.18–14.43)**	15.8	1.68 (1.15–2.46)**	9.0	5.80 (2.78–12.11)**
Parental e-cigarette use							
No	91.9	1.4	Ref	4.8	Ref	1.9	Ref
Yes	73.3	1.4	0.65 (0.22–1.93)	17.9	2.12 (1.42–3.16)**	7.4	1.91 (1.04–3.51)*
Friends’ e-cigarette use							
None	96.3	1.1	Ref	2.0	Ref	0.5	Ref
One or more	70.8	2.2	0.85 (0.40–1.80)	18.7	6.21 (4.11–9.40)**	8.4	5.28 (2.55–10.93)**

ARRR, Adjusted Relative Risk Ratios; CI, Confidence interval.

Multinomial models adjusted for all covariates.

*p < 0.05, **p < 0.01.

Model results also indicated that the likelihood of exclusive e-cigarette use was higher among adolescents who identified as gay/lesbian/bisexual/others (ARRR = 1.69, 95% CI [1.04–2.74]), lived with one (vs. both) of their parents (ARRR = 1.52, 95% CI [1.05–2.21]), had high (vs. low) FAS (ARRR = 1.71, 95% CI [1.09–2.69]), were employed (ARRR = 1.68, 95% CI [1.20–2.37]), had one or more friends who smoked (ARRR = 1.68, 95% CI [1.15–2.46]) or used e-cigarettes (ARRR = 6.21, 95% CI [4.11–9.40]), and whose parents used e-cigarettes (ARRR = 2.12, 95% CI [1.42–3.16]). Furthermore, adolescents were more likely to exclusively use e-cigarettes if they had been targeted by psychological or digital violence (ARRR = 1.55, 95% CI [1.05–2.29] and ARRR = 1.68, 95% CI [1.12–2.54], respectively).

The probability of dual-use was elevated among older adolescents (ARRR: 1.78, 95% CI [1.01, 3.14]), those employed (ARRR: 1.69; 95% CI [0.98, 2.89]) and those with one or more friends or parents who used cigarettes (ARRR: 2.03, 95% CI [1.05, 3.91] and ARRR: 5.80, 95% CI [2.78, 12.11], respectively) or e-cigarettes (ARRR: 1.91, 95% CI [1.04, 3.51] and ARRR: 5.28, 95% CI [2.55, 10.93], respectively). Furthermore, victimization by four of the five types of violence – physical, digital, neglect, and sexual – was found to be positively associated with dual use.

## 4 Discussion

This study found that experiences of violence were positively associated with exclusive use of combustible cigarettes, e-cigarettes, and dual use of both product among middle and high school students, during the COVID-19 pandemic. Experiences of physical (46.9%), psychological (42.7%), and sexual violence (5.2%) were higher than in another study of Mexican adolescents (20.3, 35.0, and 2.6%, respectively) in 2021 (32), though these differences may be due to contrasting age ranges of students (12–19 vs. 15–18) or question wording. By contrast, digital violence was higher in that other study (34.4% vs. 12.3%), perhaps because their data were collected earlier in the pandemic when students were taking classes virtually that may, in turn, have increased opportunities for exposure to online bullying (33). While previous studies have linked violence with smoking an e-cigarette use (7, 8, 34), to our knowledge, only one study in Mexico (24) has identified neglect and sexual violence associated with combustible cigarettes use, with no data e-cigarettes or dual use.

Our results indicate that adolescents who experience household physical violence were more likely to smoke, either exclusively or concomitantly with e-cigarettes (i.e., dual use). Having experienced psychological and digital violence were more strongly associated with exclusive e-cigarettes use. Additionally, dual use of combustible and e-cigarette was more prevalent among adolescents who reported experiencing four out of five types of violence: physical, neglect, digital and sexual. These finding underscore the importance of understand – and developing strategies to prevent – violent experiences, including within the family environment, as they represent stressful or traumatic precursors that seem to increase the likelihood of tobacco use (7).

Our results are generally consistent with studies of the associations between Adverse Childhood Experiences (ACEs) and cigarette use, e-cigarette use, and dual use of both products (7, 8). ACEs include physical violence, psychological violence, forms of sexual abuse and neglect, as well as living with people who have a mental illness, who suffer from substance abuse, or who are violent toward other family members (35). ACEs are also a significant factor in tobacco use-related disparities faced by sexual minority groups (34). Similarly, we found that adolescents who do not consider themselves, heterosexual are more likely to be e-cigarette and dual smokers, though not from combustion cigarettes. However, the last result may be the product of an insufficient sampling frame to identify a significant association. Future research with larger sample sizes are needed to determine whether the strength of association between experienced violence and substance use vary by sexual orientation.

Our results show that adolescents who report having suffered neglect and sexual violence are more likely to be dual users. This is consistent with a study (24) of high school students from four Mexican states that showed that males, though not females, who had suffered neglect were more likely to smoke cigarettes than those who did not. The study also measured sexual violence, finding that cigarette use is higher among adolescents who have experienced sexual violence compared to those who have not. However, the year in which this other study was conducted, e-cigarettes were not yet commercially available. To prevent substance abuse and mitigate adverse health consequences, child abuse and stress in the home likely need to be addressed.

Our results show that adolescents who are victims of digital violence – or cyberbullying – are more likely to use e-cigarettes. This may be related to the greater social media use during the pandemic. A longitudinal study (36) shows that using video calls and online shopping is related to a higher risk of starting to use e-cigarettes among adolescents, as is technophilia, whose measurement includes assessment of time spent online (21).

In Mexico, it is illegal to sell or market e-cigarettes, but it is not illegal to use them (37). Our survey revealed that a higher percentage of adolescents use e-cigarettes (8.5%) than those who smoke combustible cigarettes (3.8%). Systematic reviews and meta-analysis find that adolescents who start using e-cigarettes are four times more likely to go on to use combustible cigarettes compared to those who have not used e-cigarettes (38), although the only longitudinal study of this issue in Mexico found weaker associations than studies in other countries (39). Furthermore, Mexican adolescents who used e-cigarettes as their first nicotine product appeared to be relatively low-risk compared to those who first used cigarettes (40). This suggests that e-cigarettes help recruit people to use cigarettes (41). The government must enforce the law for tobacco control, prohibiting the sale, distribution and consumption of tobacco products, and even move toward the eliminating of flavorings in tobacco products.

Family as one of the main factors that influence health-related behaviors, such as diet, physical exercise, tobacco, and alcohol consumption (42–44). The family is also a key factor in the development of psycho-emotional protective factors. Our results show that having household rules restricting adolescent media use may protect against cigarette use, consistent with prior research on parental monitoring of adolescent behaviors (30). However, these associations were not found for adolescents who use e-cigarettes or use both products. Interpretation of these results is unclear, but it may be that specific parental rules around e-cigarettes are needed.

Our research confirms what other studies have found (21, 40, 45): adolescents from wealthier families are more likely to use e-cigarettes. This is probably because they have more access to them or the expendable income to buy them. The low cost and easy availability of cigarettes, including single cigarettes, explains the lack of association between family affluence and cigarette use.

As adolescents move through life, their decisions about smoking and using e-cigarettes are influenced more by their peers than by their parents. Our findings regarding the correlation between social and family influences among current e-cigarette users are consistent with those of previous studies (46). Current e-cigarette users and dual users are associated with having parents or friends who use e-cigarettes. However, having at least one friend who smokes increases this risk (47). Hispanic adolescents in the United States (48), have reported that e-cigarette use among their peer networks strongly influences their own decisions.

### 4.1 Strengths and limitations

Our study has some limitations. Given our cross-sectional design, we cannot determine the temporal ordering of experiences of violence and the use of nicotine products. It is unlikely, however, that nicotine product use would lead to violence, additional, unmeasured confounding variables may account for both substance use and violence in this population. Nevertheless, our models controlled for family wealth, which may be one of the socioeconomic confounders, though others may need to be explored (e.g., marginalization). The main contribution of these findings was to help reveal patterns of association between violence and substance use in a country where interpersonal violence and e-cigarette use among adolescents are growing.

## 5 Conclusion

Our results suggest a need for intersectoral interventions that likely require involvement from family, school, and the community. ACEs – which include violence of all types – are undeniably associated with addictive behaviors. Friends and parents have a significant influence, and adolescents smoking is influenced by their financial resources. This knowledge must inform the development of effective tobacco prevention strategies and legislation.

In the school context, prevention and awareness strategies must involve the active participation of parents. It is vital to emphasize their importance as role models and the behaviors they could show toward tobacco in front of their children. Prevention activities and programs may need to begin in primary education, given the relatively early age at which adolescents begin to experiment with both cigarettes and e-cigarettes.

## Data Availability

The original contributions presented in the study are included in the article/supplementary material, further inquiries can be directed to the corresponding authors.
